# Research on the Single Grit Scratching Process of Oxygen-Free Copper (OFC)

**DOI:** 10.3390/ma11050676

**Published:** 2018-04-26

**Authors:** Libin Zhang, Tao Zhang, Bicheng Guo, Lan Yan, Feng Jiang

**Affiliations:** 1 Institute of Manufacturing Engineering, National Huaqiao University, Xiamen 361021, China; libinzhang@hqu.edu.cn (L.Z.); zhangtao.ime@hqu.edu.cn (T.Z.); guobicheng@hqu.edu.cn (B.G.); 2 MOE Engineering Research Center for Brittle Materials Machining, National Huaqiao University, Xiamen 361021, China; 3 College of Mechanical Engineering and Automation, National Huaqiao University, Xiamen 361021, China; yanlan@hqu.edu.cn

**Keywords:** single grit scratching, numerical simulation, material constitutive model, material flow, friction model, residual stress distribution

## Abstract

Single grit scratching is a basic form of material removal for many processes, such as grinding single point diamond turning and coating bonding performance tests. It has been widely used in the study of micro-scale and nano-scale material removal mechanisms. In this study, single grit linearly loading scratching tests were carried out on a scratching tester. A Rockwell indenter made of natural diamond was selected as the tool used, and the material of the workpiece was oxygen-free copper. Scratch topography was measured using a super-depth microscope to analyze the material deformation of the scratching process. A single grit scratching simulation has been developed by AdvantEdge™ to comprehensively study the material deformation of scratching processes. A material constitutive model and friction model were acquired using a quasi-static uniaxial compression experiment and a reciprocating friction test, respectively. These two models were used as the input models in the finite simulations. The simulated scratching forces aligned well with the experimental scratching forces, which verified the precision of the simulation model. Since only the scratching force could be obtained in the scratching experiment, the plastic strain, material flow, and residual stress of the scratching were further analyzed using simulations. The results showed that the plastic strain of the workpiece increased with the increase in scratching depth, and further analysis showed that the workpiece surface was distributed with residual compressive stress and the sub-surface was distributed with residual tensile stress in single grit scratching.

## 1. Introduction

The single grit scratching test is used to study complex loading conditions in a controlled manner and is widely used in the study of tribology, bonding force, and workpiece material removal mechanisms. Looijmans et al. used a single grit of diamond to scratch isotactic polypropylene (iPP) at a low speed in order to study the tribological properties of iPP [1]. Jardret et al. studied the formation of car paint scratches using a quasi-static single-point scratching test [2]. The scratching test is an efficient method to characterize the cohesion of coatings on tools [3,4]. Ollendorf et al. studied the mechanical properties of TiN layers by single-point scratching and compared the results with those obtained by the surface ultrasonic method and the four-point bending test [5]. F Cao et al. studied the wear resistance of TiAlSiN coatings using single grit scratching with variable loading [6]. Sawada evaluated the fretting corrosion behavior of Ti-20Cr with different scratching speeds [7]. Chowdhury evaluated the von Mises, tensile, and shear stresses during the micro-scratch test using the Scratch Stress Analyzer [8]. Xu et al. studied the material removal mechanism of micro-cracks and crack propagation by scratching aluminum oxide with a single grit [9]. The formation of a grinding surface is a very complex process, and the single abrasive scratches experiment [10,11] is used to simplify the grinding process. J Cao et al. studied scratch morphology and grinding force variation by using a single diamond abrasive tool to scratch SiC [12]. M Zhang et al. used a single grit scratching hardened AISI 5140 steel to study the variation of scratch force at different speeds [13].

The achievements presented in the literature above only involve the results obtained by scratching experiments. However, there in a large amount of experimental unmeasurable data waiting to be discovered in scratching experiments. Finite element simulation can be used to assist in the exploration of scratching experiments. Finite element simulation is increasingly used to study the problems which cannot be measured in experiments. They can visualize complex deformation fields, such as stress and strain distributions. The constitutive model of workpiece material under quasi-static compression can be established [14] using the universal testing machine [15]. Computer aided design (CAD) software is used to establish 3D models of workpieces and tools. Then, the material constitutive model, 3D CAD models, and cutting conditions are, respectively, imported into the simulation software. Lee et al. used the finite element method to simulate steel ball scratching on glass polymer materials and studied the friction and wear mechanisms of the glassy polymeric material [16]. D Zhu et al. obtained the formation and propagation of microcracks when a single crystal diamond was scratched with different speeds and depths using numerical simulation [17]. The intergranular crack propagation of the lamellar structure of *β* titanium alloys has been investigated using finite element simulation [18]. T Zhang et al. scratched Fe–Cr–Ni with a single grit and numerically analyzed the deformation of the material and the stress distribution of the grit [19]. Li et al. used numerical simulation to analyze the temperature distribution of grinding [20]. However, the simulation research above does not introduce the experimental results, and the consistency of the simulation results and the scratching experiment results cannot be verified.

In addition, the high-temperature Hopkinson pressure bar test, quasi-static at room temperature scratching test, high-temperature quasi-static scratching test, and high-speed scratching test are the main means to study mechanical properties. The high temperature indentation experiment [21] was carried out; however, this study describes the quasi-static scratch test at room temperature. The other results will be published later. 

Oxygen-Free Copper (OFC) is widely used as an optical device [22] and material deposition substrate [23] for its good thermal and electrical conductivity. Scratching is the basic form of material removal of the micro-texture [24] on the optical device and material deposition substrate. 

Most of the studies on single grit scratching remain at the experimental stage, and all conclusions are inferred from experimental measurement data. Additionally, many other studies obtain results only through finite simulations, and the persuasiveness of their simulation results may need to be strengthened. Further exploration and improvement is needed regarding the method of single abrasive grain scratching and the scientific issues in machining. In this research, actual experimental and finite element simulations were combined. The simulation results were shown to be credible in comparison to the experimental results. The simulation results included data that cannot be measured during the actual machining experiments, such as the flow of materials, the distribution of the strain field, and the distribution of the residual stress field. Analyzing simulation data can better explain material removal mechanisms during the scratching process. 

## 2. Scratching Experiments

### 2.1. Experimental Set-Up

**Figure 1:**
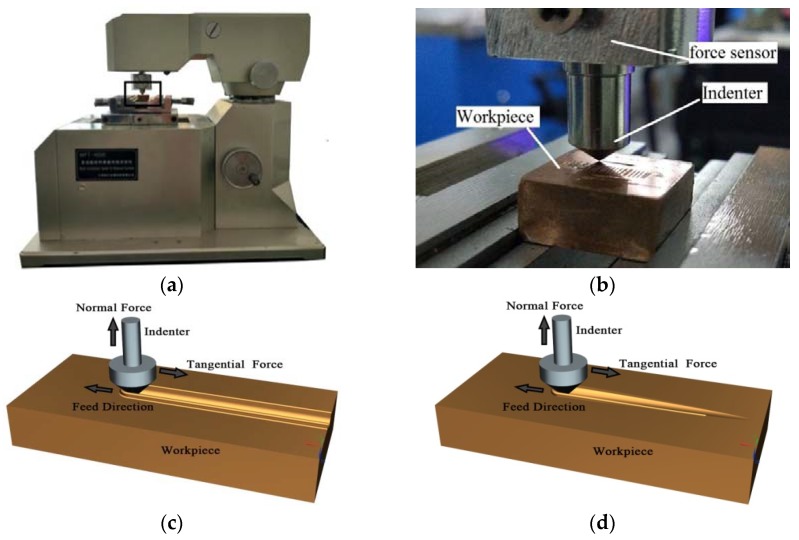
The experimental equipment and principles. (**a**) The multifunctional material surface tester; (**b**) Experimental set-up; (**c**) Sketch of constant loading scratching; (**d**) Sketch of linearly loading scratching.

The experiment was carried out on a scratching tester, as shown in Figure 1a. The tester has functions including constant loading scratching tests and linearly loading scratching tests. The workpiece was fixed on the holder and the indenter was fixed on the force sensor, as shown in Figure 1b. As shown in Figure 1c, the scratching load, scratching length, and scratching time were set at the beginning of the constant loading scratching test. The load was applied on the indenter as the normal force. The workpiece fed along a horizontal direction and the scratch was formed with a constant depth. As shown in Figure 1d, the scratching length and scratching time were set at the beginning of the linearly loading scratching test. The indenter fed evenly in the vertical direction. The workpiece started to feed evenly in the horizontal direction while the indenter contacted the workpiece. The scratching reached a final scratching depth when the workpiece fed to the set scratching length. The normal force and tangential force were recorded by a force sensor, with the sampling frequency of 200 Hz. The performance parameters of the scratching tester are listed in Table 1.

### 2.2. Workpiece and Indenter

This study involved the investigation of material flow. In order to better observe the material flow during scratching, OFC was selected as the workpiece material for its good plasticity and uniformity. The oxygen content was less than 0.003%, the total content of impurities was less than 0.05%, and the purity of the copper was more than 99.95%. The mechanical properties of OFC are listed in Table 2.

The Rockwell indenter was selected in this research. Its tip is a sphere, and the material flows along the tip surface in a simple form during the process of scratching, which can simplify the scratching process. Thus, the Rockwell indenter is suited to research the uniform deformation behavior of materials and is widely used in scratching experiments [3,5,21,25]. It is also suitable for the study of copper [26]. The indenter was made of natural diamond, the properties of which are listed in Table 3. The cone angle was 120° and the tip radius was 0.2 mm.

### 2.3. Scratching Parameters 

Linearly loaded scratching was applied in this study. The final scratching depth was set to 75 μm. The scratching length was set to 6 mm, and the scratching time was set to 60 s. The scratching tests were repeated three times.

### 2.4. Measurement of Scratching Topography

**Figure 2:**
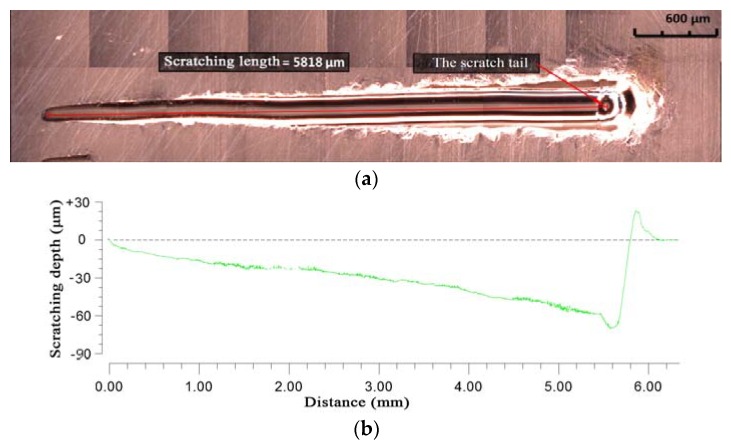
The measurement results of scratching. (**a**) Full picture of the scratch; (**b**) The actual scratching depth of the scratch.

The scratch topography was measured using a super-depth microscope and Zygo NV7300 white light interferometer (WLI), respectively. The results are shown in Figure 2. The full topography of the scratch could be acquired using the super-depth microscope, as shown in Figure 2a. The actual scratching depth of the scratch could be measured by WLI, as show in Figure 2b. The average scratching length according to the measured results was 5.88 mm, which was smaller than the set scratching length. The reason for this is that there is no scratch formation when the scratching depth is extremely small, which is called the sliding stage. The average final scratching depth, according to measured results, was 72.2 μm, which was smaller than the set final scratching depth due to the elastic rebound of the workpiece material. By calculating the average length of the scratches and the average maximum depth, the slope angle at the bottom of the scratches was found to be 0.703°.

## 3. Numerical Simulation

**Figure 3:**
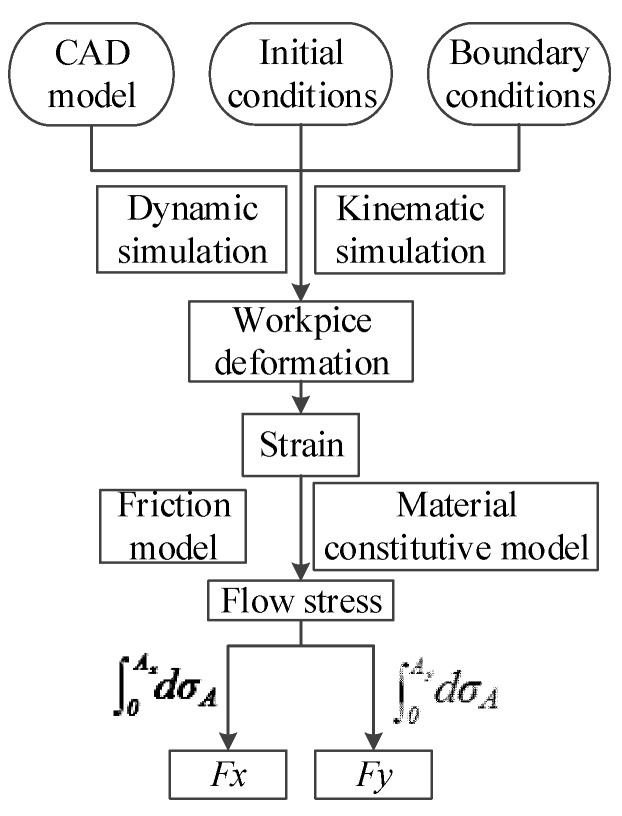
The procedure of numerical simulation.

The commercial finite element method (FEM) code AdvantEdge™ was used in the scratching simulation. This is powerful finite element analysis software that allows users to customize the conditions and parameters of the entire simulation process. A number of physical quantities can be obtained by the simulation result, and some of them, such as stress distribution, strain distribution, and material flow, are impossible to acquire by experiments. The procedure of numerical simulation is shown in Figure 3. The CAD model of the indenter and workpiece, the initial conditions (such as the initial temperature), and the boundary conditions (such as the degree of freedom workpiece and indenter) were required as inputs. The workpiece material constitutive model and friction model were also required as inputs, as they are necessary for the simulation. AdvantEdge™ provides many simulation types for users and, in this study, the grooving process type was selected. 

Because of the fairly low scratching speed, thermal softening and strain rate hardening were ignored. The absence of the thermo-mechanical coupling phenomenon was due to the ignoring of thermal softening. The deformation of workpiece geometries was calculated from dynamic and kinematic simulations. By comparing the initial and the deformed geometry of the workpiece, the strain was calculated. With the constitutive model and strain, the normal stress between the workpiece and the tool could be calculated. Sheer stress can be calculated by connecting the normal stress and friction models. The flow stress of the workpiece material can be calculated from the normal stress and sheer stress. Finally, the scratching forces were calculated by the integration of the flow stress at the contact area. 

### 3.1. 3D Simulation Model

AdvantEdge™ adopts the Lagrangian finite element model that is based on adaptive tetrahedral meshing. The workpiece and the indenter imported into AdvantEdge™ need custom grid generation. The custom parameters include maximum element size, minimum element size, mesh grading, curvature safety, segments per edge, and minimum edge length. The maximum element size defines the size of the largest cell in the grid. The minimum element size defines the size of the minimum cell in the grid. The mesh grading determines the nature of transition from fine elements. If the value is close to 0.1, this will lead to very slow transition results in the overall refined mesh. If the value is close to 1, this will lead to fast transition results in coarse mesh. Curvature safety determines the accuracy with which curved geometric features in the model are captured in the mesh. The segments per edge are defined as the density of the nodes on any edge length. The value of curvature safety and the segments per edge from 0.2 to 5.0 correspond to the mesh, from coarse to fine. The minimum edge length determines the minimum allowable edge length for any element in the mesh. The mesh parameters are listed in Table 4.

**Figure 4:**
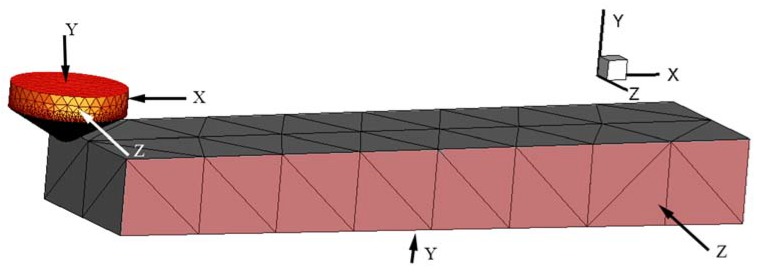
3D simulation model and boundary conditions.

The 3D CAD model and boundary conditions of the workpieces and tools are as shown in Figure 4. The size and shape of the tool in the model was a Rockwell indenter, stated in 2.2. The workpiece CAD model was a cuboid, and the dimensions were 8 mm × 2 mm × 1 mm. The workpiece was rotated 0.703° around the *Z* axis which corresponds to the experiment results. In this model, the indenter was fixed in three directions and the workpiece was fixed in *Y* and *Z* directions and could only feed along the *X* direction.

One feature of AdvantEdge™ is that the grid of the workpiece close to the grit is automatically refined and the grid away from the grit is coarsened during the simulation, which is called adaptive meshing. This feature takes both simulation efficiency and simulation accuracy into consideration. The parameters that were used to control the meshing process of the workpiece included the minimum edge length (chip bulk and cutter edge), set to 0.014 mm, and the refined region radius, set to 0.008 mm. The minimum edge length was set to half of the default value and the refined region radius was twice the default value. As for the indenter, defining the element size could refine it. The element size was set to 0.01 mm at the indenter tip.

### 3.2. Material Constitutive Model and Fiction Model 

#### 3.2.1. The Material Constitutive Model

The scratching speed in this study was extremely slow. The oxygen-free copper was squeezed by the indenter in the scratching process. Therefore, the material constitutive model for the simulation was able to be developed by a quasi-static experiment. The quasi-static compression experiment was carried out in Instron 8874, and the experiment design is outlined in Table 5.

**Figure 5:**
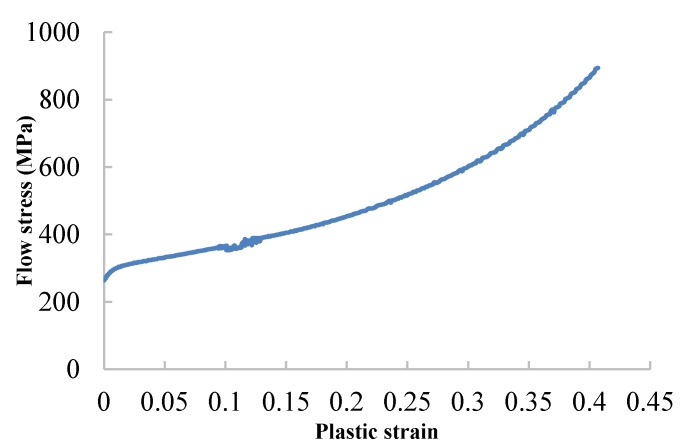
Flow stress-plastic strain curves.

The true stress-strain curve, which was transformed from the engineering stress-strain curve, is shown in Figure 5.

At present, the Johnson–Cook constitutive model [19,27] and power-law [21] constitutive model are commonly used for material constitutive relations.

The Johnson–Cook model, as a constitutive model in the process of material processing, describes the elastoplastic behavior of a material. It mainly considers the strain rate effect and temperature effect, but also considers large strain, high strain rate, suitability for simulation, and strain-related large deformation problems.

The three parts of the model, from left to right, describe the strain hardening effect of the material, reflect the relationship between the flow stress and the logarithmic strain rate, and reflect the relationship between the flow stress and the temperature increase index, respectively. The equation is as follows:

(1) $$\bar{\sigma }=\left(A+B{\left(\varepsilon \right)}^{n}\right)\left(1+C\ln\left(\frac{\dot{\varepsilon }}{{\dot{\varepsilon }}_{0}}\right)\right)\left(1-{\left(\frac{T-T_{0}}{T_{melt}-T_{0}}\right)}^{m}\right)$$

where $\bar{\sigma }$ is material flow stress; *ε* is plastic strain; $\dot{\varepsilon }$ is the strain rate (*s*^−1^); ${\dot{\varepsilon }}_{0}$ is the reference plastic strain rate (*s^−^*^1^); *T* is the workpiece temperature (°C); *T_melt_* is the workpiece material melting temperature (°C); *T*_0_ is room temperature (20 °C); *A* is the yield strength (Mpa); *B* is the modulus of elasticity (MPa); *C* is the rate of strain sensitivity; *n* is the coefficient of hardening; and *m* is the coefficient of thermal softening.

However, the constitution of this study was established for AdvantEdge FEM simulation software. This software mainly uses the power-law model. The form of the power-law model is simple, and the use of variables are applicable to many kinds of computer codes. In conclusion, it is a practical model that can be applied to analysis and calculation. This study used the power-law model to establish the constitutive model. 

There are strain hardening effects, strain rate strengthening effects, and heat softening effects in metal deformation. The strain hardening effect refers to material flow stress increasing with increasing strain. The strain rate hardening effect refers to material flow stress increasing with the increasing strain rate. The thermal softening effect refers to material flow stress decreasing with increasing temperature.

The basic expression of the power-law model is:

(2) $$\sigma \left(\varepsilon^{p},\dot{\varepsilon },T\right)=g\left(\varepsilon^{p}\right)\cdot \Gamma \left(\dot{\varepsilon }\right)\cdot \Theta \left(T\right)$$

where *g*(*ε^p^*) is the strain hardening effect; $\Gamma \left(\dot{\varepsilon }\right)$) is the strain rate enhancement effect; and Θ(*T*) is the thermal softening effect.

The strain hardening effect is defined as:

(3) g(εp)=σ0(1+εpε0p)1n

where *σ*_0_ is the yield stress at reference strain; *ε^p^* is the strain; $\varepsilon_{0}^{p}$ is the reference strain; and *n* is the strain hardening factor.

The strain rate hardening effect is defined as:

(4) Γ(ε˙)=(1+ε˙ε˙0)1m

where $\dot{\varepsilon }$ is the strain rate; ${\dot{\varepsilon }}_{0}$ is the reference strain rate; and *m* is the strain rate enhancement factor.

The thermal softening effect is defined as:

(5) $$\left\{ \begin{array}{ll} \Theta \left(T\right)=c_{0}+c_{1}T+c_{2}T^{2}+c_{3}T^{3}+c_{4}T^{4}+c_{5}T^{5} & T<T_{cut}\\ \Theta \left(T\right)=\Theta \left(T_{cut}\right)\left(1-\frac{T-T_{cut}}{T_{melt}-T_{cut}}\right) & T>T_{cut} \end{array}\right.$$

where *c*_0_–*c*_5_ are polynomial fitting coefficients; *T* is the processing temperature; *T_cut_* is the linear softening starting temperature; and *T_melt_* is the melting point of the material.

In this study, strain rate hardening and thermal softening can be ignored because of the fairly low strain rate and the little temperature rise in the experiment Then, the simplified material constitutive model based on the power-law relationship can be expressed as:

(6) σ=σ0(1+εsε0)1n

where *σ* is the flow stress.

The constant *σ*_0_ can be measured from the start point of the plastic strain-flow stress curve. The stress at the plastic strain was 0. The constant *σ*_0_ was measured as 263.33 MPa. Two sides of the Equation (6) were divided by *σ*_0_. Then, taking the logarithm of both sides, the equation could be expressed as the following:

(7) $$\ln\frac{\sigma }{\sigma_{0}}=\frac{1}{n}\cdot \ln(1+\frac{\varepsilon_{s}}{\varepsilon_{0}})$$

**Figure 6:**
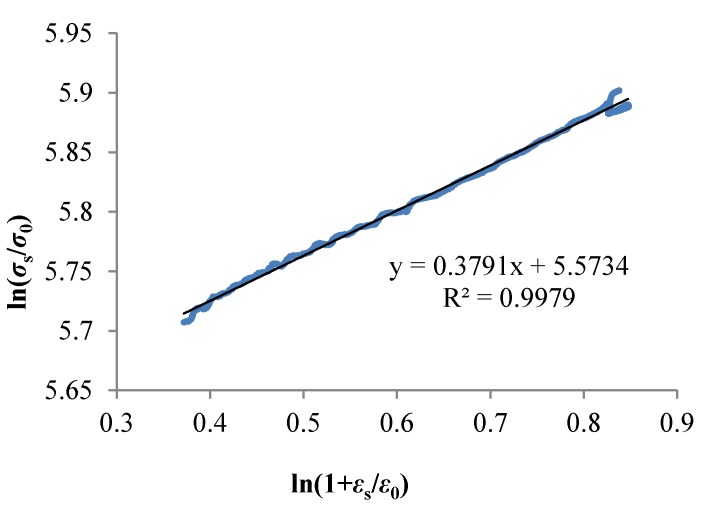
The relationship between ln(*σ*_s_/*σ*_0_) and ln(1 + *ε*_s_/*ε*_0_).

The reference strain *ε*_0_ was taken as 0.1 and the flow stress of the plastic segment and its strain were taken from Equation (7). Then, using the least square method to fitting the curves, as shown in Figure 6, the result showed that *R^2^* was 0.9979 and the slope (*1*/*n*) of the line was 0.3791. The strain hardening factor (*n*) was 2.638.

#### 3.2.2. The Friction Model

**Figure 7:**
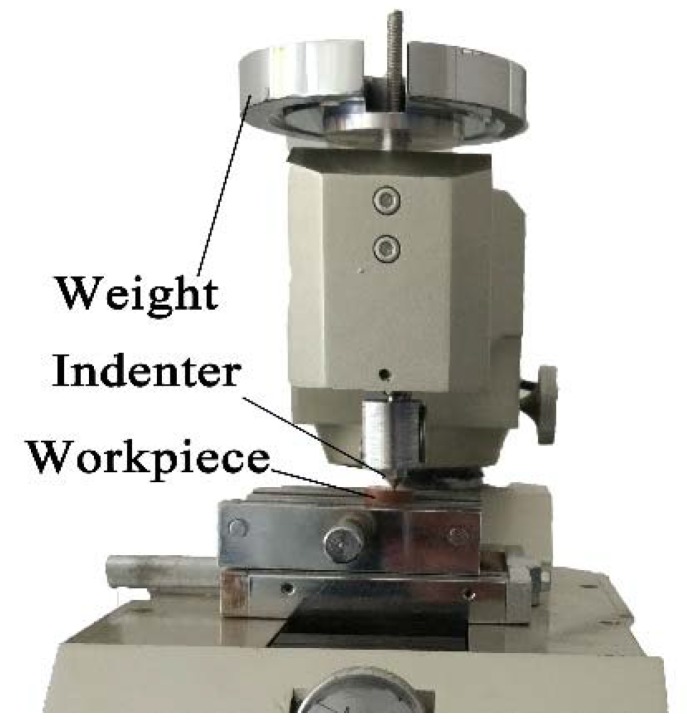
Experimental set-up of the friction coefficient.

**Figure 8:**
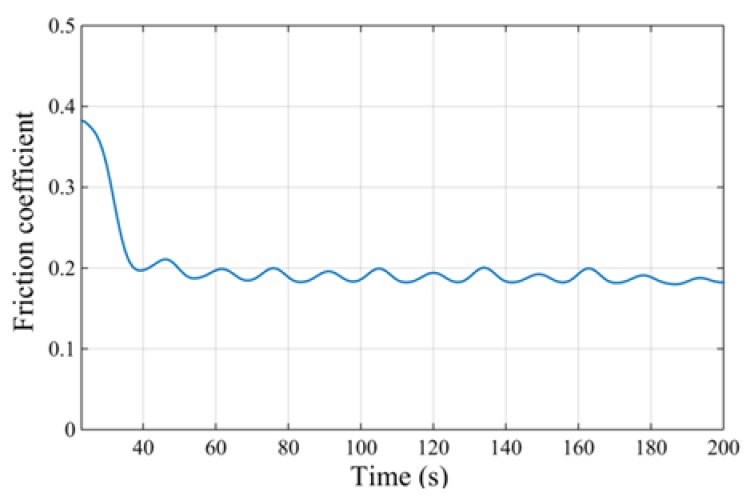
The friction coefficient curve.

Measurement of the friction coefficient between the OFC and the diamond indenter was performed on a multifunctional surface performance tester. The workpiece was fixed on a fixture, and the indenter scratched on the surface of the workpiece reciprocally. The steady load on the indenter was provided by its weight, as shown in Figure 7. The scratching speed was extremely low, which can be seen as a quasi-static scratching process. The test results showed that the friction coefficient between the OFC and natural diamond was 0.19, as shown in Figure 8.

#### 3.2.3. Other Input Paraments

In this simulation, the indenter material was defined as a diamond (nature/single crystal), and the other parameters of the workpiece material were entered manually. The thermal conductivity and heat capacity of the OFC was 387 W/(m°C) and 385 J/(kg°C), respectively. The density was 8900 kg/m^3^, Young’s modulus was 1.17 × 10^11^ Pa, and Poisson ratio was 0.31.

## 4. Results and Discussion

### 4.1. Scratching Forces and Force Ratio

**Figure 9:**
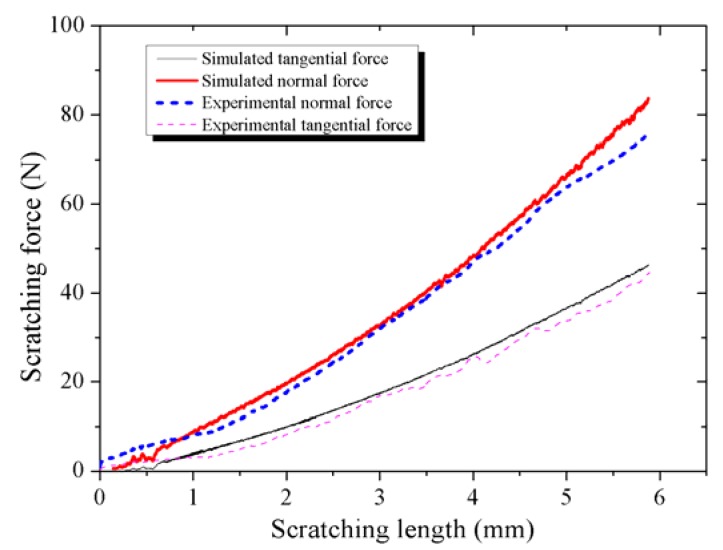
Comparison of the scratching forces acquired by the simulation and experiment.

In this study, the force data collected by the multi-functional material surface performance tester and obtained by AdvantEdge™, are compared in Figure 9. The simulated forces aligned with the experiment results, which verifies the simulation precision.

It is impossible to completely simulate the actual scratch process. Finite element simulation subdivides the object into a finite number of units. The smaller the unit, the higher the simulation precision, but this also results in a greater number of units, resulting in an increase in the simulation calculation. The simulation in this study was carried out after balancing the power of the computer and the error. 

The friction model also increased errors. The actual friction between the workpiece and the tool was complex, and this simulation simplified the friction model to an average friction coefficient. The constitutive model of the workpiece material ignored strain rate strengthening and thermal softening, too. In addition, the software algorithm was a source of error. All of these factors led to error in the simulation result, but it can be inferred from Figure 9 that this error is acceptable.

### 4.2. Plastic Strain Distribution of the Workpiece

**Figure 10:**
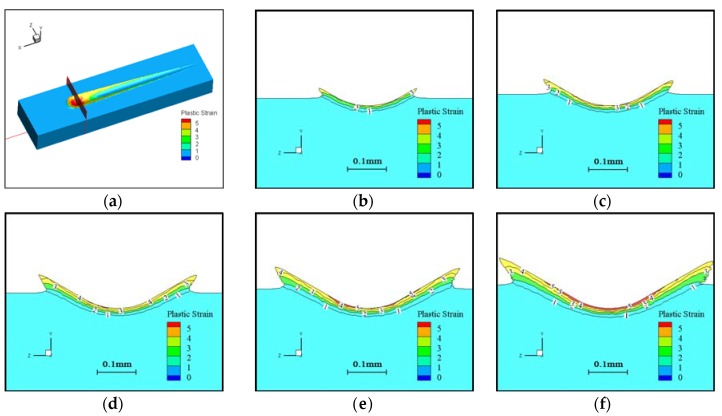
The plastic strain distribution along the scratch under different scratching lengths. (**a**) Plastic strain distribution along the scratch; (**b**) Length of scratch = 2 mm; (**c**) Length of scratch = 3 mm; (**d**) Length of scratch = 4 mm; (**e**) Length of scratch = 5 mm; (**f**) Length of scratch = 6 mm.

In the scratching process, scratching depth increased with scratching distance. In the simulation, the plastic strain distribution of the workpiece is shown in Figure 10. Figure 10a shows a schematic cross-sectional view of Figure 10b–f, and Figure 10b–f shows the plastic strain distribution of the cross-sections of different lengths. It can be seen from Figure 10 that the plastic strain of the workpiece increased with distance, which is consistent with the scratching expiration.

### 4.3. Material Flow 

**Figure 11:**
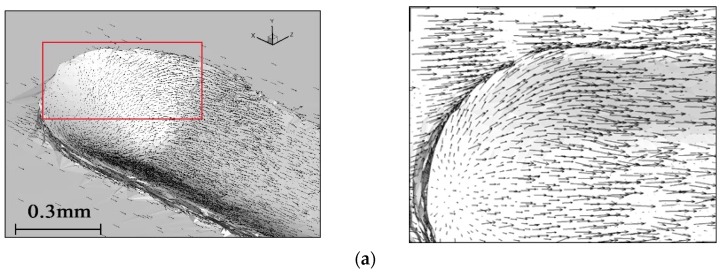

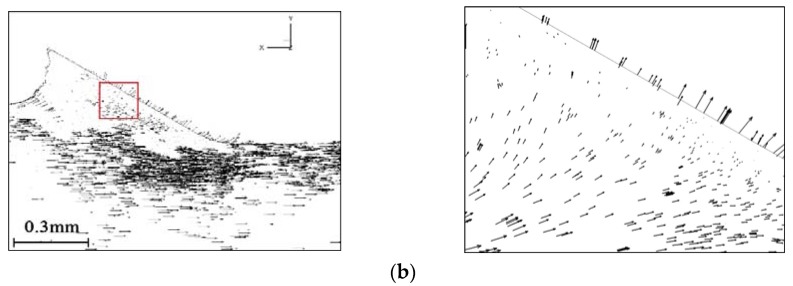
The material flow and accumulation (length of scratch = 6 mm). (**a**) Partial view; (**b**) Longitudinal section view.

**Figure 12:**
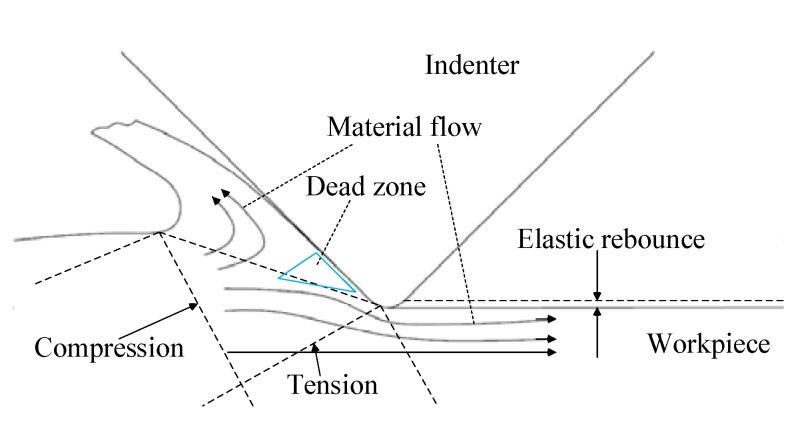
Schematic diagram of the dead zone.

It is difficult to measure material flow during a scratching experiment, but simulation makes it possible. Figure 11 shows the flow of workpiece material relative to the indenter. The direction and length of the arrows indicate the direction and velocity of the material flow. As seen in Figure 11a, the workpiece material in front of the tool formed an accumulation, and the material flow rate was very low, even stopping. This was because the workpiece material was squeezed by the tool and had to flow upward, downward, or sideways. There was an equilibrium position in the material flow area. The workpiece material flowed at a velocity close to zero, forming a dead zone, as shown in Figure 11b and Figure 12. The formation of a dead zone is associated with the geometry of the tool. A larger rake angle or sharper blade will make the material flow easier in the process and reduce dead zone size [28]. In addition, the better the plasticity of the workpiece material, the larger the size of the dead zone [22]. The dead zone is located in front of the indenter and works as the indenter scratches into the workpiece. 

**Figure 13:**
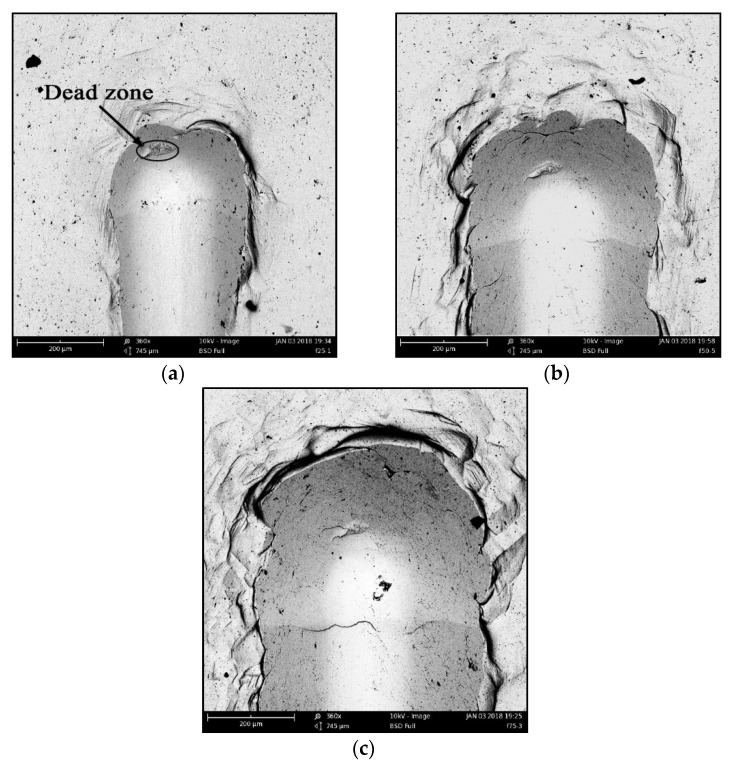
The topography of scratches at different depths. (**a**) Scratching depth = 25 μm; (**b**) Scratching depth = 50 μm; (**c**) Scratching depth = 75 μm.

Figure 13 shows the topography of scratches at different depths taken by a scanning electron microscope (SEM). It is obvious that a dead zone exists from the material accumulation forming in the process of scratching.

### 4.4. Residual Stress Distribution

**Figure 14:**
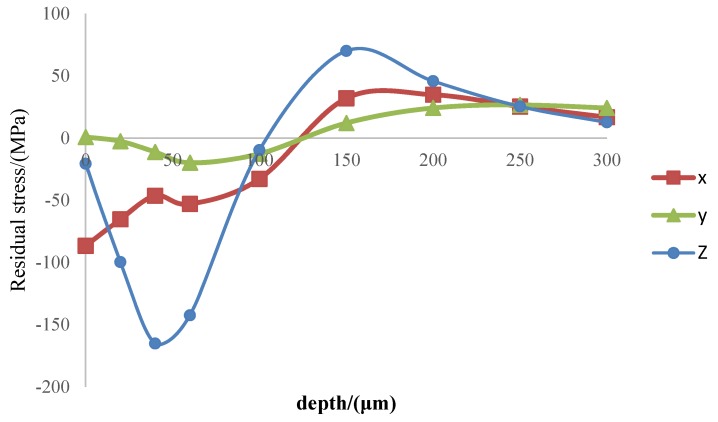
Residual stress (scratching length = 5 mm).

Figure 14 shows the distribution of residual stress in the depth direction at a scratching distance of 5 mm in the simulation results. It can be seen that the residual stress in the workpiece surface was compressive stress, and that it was tensile stress in the workpiece sub-surface.

In the scratching process, residual stress was closely related to mechanical deformation. The scratching force of the indenter caused plastic deformation on the workpiece surface and sub-surface. In the process of squeezing forward, the sub-surface material was stretched by the accumulation, but the indenter had a squeezing effect on the surface, as shown in Figure 12. In the process of single grit scratching, a part of the material flowed along the front of the indenter and formed an accumulation. A part of the material was squeezed by the indenter tip and flowed to the back of the indenter, forming the processed surface. Therefore, compressive stress was generated in the machined surface. In the formation of the processed surface, the pressed material partially rebounded and the residual compressive stress decreases. 

## 5. Conclusions

1. A single grit scratching test and quasi-static compression experiments of OFC were carried out. A constitutive model of the compression experiment was input into the AdvantEdge^TM^ cutting finite element simulation software to carry out the single grit scratching numerical simulation. The simulation forces aligned well with the experimental results.
2. In the simulation, the plastic strain of the workpiece increased with scratching depth. This result also aligns with the actual situation.
3. The material in front of the tool flowed upward and sideways, and there was an equilibrium position in this area, where the material flow rate was zero and formed a material dead zone. The dead zone material worked as an indenter, scratching into the workpiece.
4. The distribution of residual stress on the processed surface of the single grit quasi-static scratching was compressive stress on the surface and tensile stress on the sub-surface.
5. It was difficult to measure data inside the material. Stress field, strain field, strain rate field, and temperature field distribution cannot be measured experimentally; however, a simulation can visualize the material interior. A highly accurate simulation result can describe the results of the scratching test better. In addition to normal force and tangential force, it can show the stress field, strain field, strain rate field, and temperature field distribution inside the material.

## Figures and Tables

**Table 1:** The fundamental performance parameters of the tester.

Fundamental Performance	Range
Load range (N)	0–200
Tangential force range (N)	0–100
Loading speed (N/min)	10–100
Scratch length (mm)	2–50

**Table 2:** The mechanical properties of Oxygen-Free Copper (OFC) stainless steel.

Mechanical Parameters	Measured Value
Tensile strength, ultimate	455 MPa
Elongation	55%
Poissons ratio	0.31
Forgeability	65%

**Table 3:** The properties of natural diamond.

Properties	Measured Value
Density	3.52 g/cm^2^
Hardness, Mohs	10
Modulus of elasticity	1200 GPa
Compression strength	16,530 MPa
Poisson’s ratio	0.29
Specific heat capacity	0.5079 J/g °C
Thermal conductivity	2000 W/mK

**Table 4:** Parameters related to the mesh process.

	Parts	Workpiece	Indenter
Parameters			
Maximum element size/mm		1	1
Minimum element size/mm		0.1	0.1
Mesh grading		0.5	0.5
Curvature safety		1.5	1.5
Segments per edge		0.5	0.5
Minimum edge length/mm		0	0.002

**Table 5:** Design of the experiment for uniaxial compression.

Items	Parameters
Sample size (mm)	Ø6 × 5 (Cylinder)
Compression speed (mm/min)	1
Insulation time (min)	15
Test temperature (°C)	18
Humidity (%)	50
